# New Horizons for Plant Translational Research

**DOI:** 10.1371/journal.pbio.1001880

**Published:** 2014-06-10

**Authors:** Jane Alfred, Jeffery L. Dangl, Sophien Kamoun, Susan R. McCouch

**Affiliations:** 1 *PLOS Biology*, Public Library of Science, Cambridge, United Kingdom; 2 Howard Hughes Medical Institute, University of North Carolina, Chapel Hill, North Carolina, United States of America; 3 Department of Biology, University of North Carolina, Chapel Hill, North Carolina, United States of America; 4 The Sainsbury Laboratory, Norwich, United Kingdom; 5 Department of Plant Breeding and Genetics, Cornell University, Ithaca, New York, United States of America

## Abstract

In this issue, we launch a new article collection "The Promise of Plant Translational Research," featuring articles from leading plant researchers and call for additional plant translational research to be submitted to *PLOS Biology* for inclusion in this collection. We also discuss in this Editorial why this field has a vital role to play in meeting the challenges of sustainably feeding a growing world population.

This article is part of the *PLOS Biology* Collection “The Promise of Plant Translational Research.”

The world's human population continues to expand and is predicted to reach ∼9 billion by 2040, up from its current level of just over 7 billion [1],[2]. Some estimate that with this rate of population growth, accommodating the increased demand for food will require the world's agricultural production to increase 50% by 2030 [3]. The planet's water resources are also under pressure. As Pamela Ronald highlights in her accompanying Essay [4], the amount of fresh water available per person has decreased 4-fold in the last 60 years and of the water that is available, ∼70% is already used for agriculture [5]. Thus, agricultural production must be intensified to feed more people with less water on the same amount of land (given that little undeveloped arable land remains and what does is being lost to urbanization, desertification, and environmental damage [5]). Furthermore, pathogens that cause devastating crop losses continue to spread in the face of increased global commerce and climate change [6]. Given these challenges, there is a pressing need for plant research to produce solutions to ensure food security in a sustainable and safe way. The need is acute in both developed countries and in the less developed parts of the world, where many people endure chronic malnutrition and suffer the long term consequences on their health and well being. Plant scientists, therefore, urgently need to increase the productivity, pathogen resistance, and sustainability of existing crops, and are challenged to domesticate new crops [7].

Plant translational research—the development of basic plant research discoveries into technologies or approaches that improve agriculture—has a vital role to play in meeting these challenges. We felt the time was therefore right to highlight this important field of research in *PLOS Biology* in the form of a collection of articles contributed by leaders in this field [4],[9]–[12] and to call for additional research articles to be submitted to *PLOS Biology* on this topic (see Box 1 for more information on this collection and the accompanying call for papers). The technical advances highlighted in this collection, for example, in genome editing [8], in plant metabolic engineering [9], and in next generation sequencing [10], exemplify how basic research discoveries are being translated into methods to develop and improve, both agriculturally and environmentally, important crop traits. Such improvements matter because they can increase food production per unit of water and hectare of land, reduce the amount of pesticides used to protect crops from pests and diseases, and enhance the nutritional content of what is grown [4],[9]. A reduction in the amount of crops lost to pests, pathogens, and environmental stresses would have the same impact as creating more land and fresh water [3],[4],[13],[14].

**Box 1. *PLOS Biology* Collection: The Promise of Plant Translational Research**

In this Issue of *PLOS Biology*, we launch a new collection, entitled “The Promise of Plant Translational Research.” This collection features a selection of Essay and Perspective articles contributed by leaders in the plant research field [4],[8]–[12], on topics ranging from technological advances [8]–[11], to discussions of key societal and regulatory issues, in plant breeding [4],[12]. The collection also features relevant research articles previously published in *PLOS Biology* and other PLOS journals.

We call for additional plant research that has clear translational possibilities to be submitted to *PLOS Biology* for inclusion in this collection. Research articles that are submitted in response to this call for papers by May 31, 2015 and published by *PLOS Biology* will not incur an author publication charge. Our usual editorial criteria for publication will apply. When submitting research articles to be considered for inclusion in the collection, please state in your cover letter and submission form that the submission is intended for The Promise of Plant Translational Research Collection (please contact plosbiology@plos.org if you have any queries regarding submission).

*PLOS Biology* thanks our advisory Academic Editors for their advice on this collection: Jeff Dangl, Sophien Kamoun, and Susan McCouch. We are also grateful to the Bill & Melinda Gates Foundation for their support of this collection. The Foundation has no control over the content, or the selection of articles, for this collection beyond identifying its general subject area and wishing to pay tribute to the work of Simon Chan, whose work the Foundation funded before Simon's untimely death in 2012. As his former colleague Luca Comai recounts in his accompanying Perspective article [11], Simon Chan recognised how a basic research discovery could be developed into a potential tool for plant breeding, leaving a legacy of work that holds future promise for improving, among other efforts, the breeding of clonally propagated staple food crops.

However, nascent translational plant biology itself faces some very real challenges. Over the past two decades, there has been a considerable reduction in the public funding of basic plant biology research, which underpins translational agricultural research. This decreased public funding has been accompanied by an unprecedented growth in agricultural research and development in the private sector—a trend that is particularly noticeable since the 1990s in wealthy countries [15]. This shift in funding patterns has profoundly impacted the type of agricultural research that is undertaken globally. With a strengthening of intellectual property (IP) rights, restrictions on the exchange of germplasm, and introduction of costly biosafety regulations, the private sector increasingly focuses on the development of crop technologies that are likely to maximize profits, and prefers to extend or expand the use of existing products rather than take the risk of translating basic research discoveries into new products [16]. A result of this behaviour is a growing gulf between basic and applied research, a gap that could be filled in the public sector by renewed support for both basic and translational research and in the private sector by a more robust start-up environment focused on translational agricultural research. At the same time, investments in public breeding efforts and plant breeding education have been drastically reduced, resulting in a paucity of new inbred and open-pollinated varieties of vegetable and staple food crops world-wide, and a significant shortage of well-trained, professional plant breeders [17],[18].

One way in which the gap between public research funding and corporate product development is being filled is through public-private partnerships. Foundations involved in such partnerships include the Rockefeller Foundation [19], the Bill & Melinda Gates Foundation [20], the Gatsby Foundation [21], and the Howard Hughes Medical Institute in collaboration with the Gordon and Betty Moore Foundation [22]. These foundations support specific projects and, in some instances, research institutes, to sustain wider ranging research programs. The long-term research promoted by these foundations has resulted in important basic discoveries being made in plant epigenetics, immunity, pathogen genomics, plant-environment interactions, structural biology, and metabolic engineering; discoveries that have laid the foundation for a number of translational applications. The recognition that basic discoveries made in plant biology are of profound importance to human health drove this investment [23]. However, while these partnerships are important, they do not replace the broad-based, public-sector funding that drives a rich discovery pipeline, nor can they replace the dynamic tension created by a well-funded innovation ecosystem.

New technologies, including high-throughput DNA sequencing, advanced mass spectrometry, parallelized computing, remote sensing, and diverse forms of information technology are also having a profound impact on translational agricultural research, opening the door to new kinds of collaborations and new avenues for information exchange. Social media is being used to monitor pests, diseases, and climatic conditions, making it possible to incorporate real-time information into translational research networks that are increasingly decentralized and international. These advances also create new opportunities for large-scale modelling and prediction. Models can be created on-the-fly from large datasets streamed from genome sequencing centers and can help to set priorities in translational research to increase the efficiency of plant breeding, to manage pests and diseases, to increase “resource use efficiency,” and to develop resilient crop varieties and cropping systems tailored to specific environments and ecologies. Technological improvements are also aiding farmers, by providing them with important information about what to plant, and about how to manage and market their crops, and through the provision of new kinds of crop insurance policies that seek to lower the risk of early adoption of innovation on the farm. With renewed interest in making agriculture more sustainable and productive, and a better ally in promoting human health, there are many exciting opportunities for a new generation of plant scientists to come forward with fresh ideas and novel approaches to enliven and embolden the translational research community.

At *PLOS Biology*, we are committed to supporting breakthroughs in both basic and translational plant science. We encourage plant researchers to submit their high quality plant research to *PLOS Biology*, and in particular, plant research that has clear translational possibilities (see Box 1). Given the importance of research in this field, we believe strongly that such work should be published in open access journals, ensuring that it reaches the widest possible audience without any barriers to access, published under a CC-BY licence, and with associated data made accessible and reusable. Open access publishing of plant research should be an integral part of the scientific community's response to achieving food security and food safety for all of the planet's human population, using environmentally sustainable approaches.
